# αMSH inhibits adipose inflammation via reducing FoxOs transcription and blocking Akt/JNK pathway in mice

**DOI:** 10.18632/oncotarget.17465

**Published:** 2017-04-27

**Authors:** Guannv Liu, Meihang Li, Muhammad Saeed, Yatao Xu, Qian Ren, Chao Sun

**Affiliations:** ^1^ College of Animal Science and Technology, Northwest A&F University, Yangling, Shaanxi, 712100, China

**Keywords:** αMSH, adipose, inflammation, FoxOs, Akt/JNK

## Abstract

Alpha melanocyte stimulating hormone (αMSH) abates inflammation in multiple tissues, while Forkhead box proteins O (FoxOs) stimulate inflammatory cascade. However, the relationship between αMSH and FoxOs in adipose inflammation remains unclear. In this study, we used LPS-induced inflammation model, attempted to interpret the function of αMSH in inflammation and the interactions with FoxOs. Results indicated that upon inflammatory situation, the secretion of αMSH and the expression of its receptor MC5R were greatly decreased, but FoxOs expressions were elevated. After the treatment with αMSH, LPS-induced adipose inflammation together with FoxOs expressions was significantly reduced. Conversely, when Foxo1, Foxo3a or Foxo4 overexpressed in αMSH treated inflammatory mouse model, all the anti-inflammatory impacts of αMSH were found disappeared. We further studied the mechanisms by which αMSH exerts its anti-inflammatory impacts and how FoxOs reverse αMSH's function. Foxo4 was found as a negative regulator for MC5R transcription in αMSH inhibited inflammation. Moreover, a negative role was found of αMSH in regulating both Akt and JNK signal pathways by observing the enhanced the anti-inflammatory impacts of pathway-specific inhibitors with αMSH treatment. Our findings demonstrate αMSH plays a key role in the prevention of adipose inflammation and inflammatory diseases by down-regulating Akt/JNK signal pathway and negatively interacting with FoxOs, which brings up αMSH as a novel candidate factor in the adipose anti-inflammation process in obesity.

## INTRODUCTION

Obesity and Type 2 diabetes are accompanied with chronic inflammation, which are involved in many disorders and diseases in human [1, 2]. Chronic inflammation in adipose tissues promotes the development of ectopic fat deposition, and is essential for healthy adipose tissue expansion and remodeling [3, 4]. Excess lipid deposition exactly contributes to immune cell proliferation, pro-inflammatory cytokines releases, macrophage accumulation in adipose tissue, perivascular adipose tissue disturbance, acceleration of subsequent macrophage infiltrating into white adipose tissue, and susceptibility to obesity, insulin resistance or Type 2 diabetes [5–10]. Fatty acid synthesis is also considered to be indispensable for adipose chronic inflammation [11]. Recent studies show that adipose tissue inflammation and fibrosis are the pathological process linking to obesity, and external stimuli hypoxia and free fatty acids exacerbate inflammation [12, 13]. M1 macrophages release pro-inflammatory cytokines that result in insulin resistance, while M2 macrophages secrete anti-inflammatory cytokines to protect against obesity and inflammation, thus macrophage polarization status control immune process [14–16]. Therefore, deciphering the molecular mechanism of adipose inflammation leads to development of a novel therapy for human inflmmatory diseases, such as obesity-induced insulin resistance and diabetes.

Alpha melanocyte stimulating hormone (αMSH), is produced by proopiomelanocortin (POMC) neuron, which is one of neuronal populations of arcuate nucleus (ARC) in brain. αMSH reduces food intake and energy storage, and increases energy expenditure of the peripheral tissues and organs via interacting with its receptors [17, 18]. As an endogenous neural immunomodulatory peptide, αMSH is involved in nerves system-endocrine-immune network. αMSH or its analog inhibits nitric oxide (NO) production, and reduces lipopolysaccharide endotoxin (LPS) or TNFα induced inflammation [19]. Previous studies have showed that αMSH abolishes tumor necrosis factor α (TNF α)-induced NF-κB activation in nasal epithelial cells, and inhibits hypothalamic inflammation response to IL-1β as well [20, 21]. LPS has been shown to induce inflammation in adipocytes, myocytes and hepatocytes [19, 22]. αMSH is obviously resistant to inflammatory response, and it blunts LPS-induced inflammation by controlling NF-κB activation and Akt/FoxO1 pathway in skeletal muscle [23]. αMSH in adipose tissue is known for its antioxidant and anti-inflammatory properties, which can abate LPS-induced inflammation and obesity [24, 25]. Previous studies in our lab revealed that αMSH accelerates preadipocyte proliferation by alleviating ER stress-induced Leptin resistance, and it promotes fatty acid oxidation in adipose tissues together with transcription factor Foxc2 [26, 27]. In addition, transcription factors Foxo1, Foxo3a, Foxo4 and Foxo6 in mammals have been found to promote inflammatory cascades in adipose tissues and maintain immune system homeostasis [28–31]. However, whether the transcription factors FoxOs are involved in αMSH exerting pivotal effects on adipose inflammation and the molecular mechanism remain to be elucidated.

In this study, we demonstrated that αMSH inhibited LPS-induced adipose inflammation. Moreover, we showed that FoxOs transcriptional inhibition and phosphorylation blocking of Akt/JNK signal pathway were involved in αMSH inhibited adipose inflammation. The results might serve as theoretical basis for inflmmatory diseases.

## RESULTS

### αMSH attenuates LPS-induced adipose inflammation by inhibiting FoxOs expressions in mice

**Figure 1:**
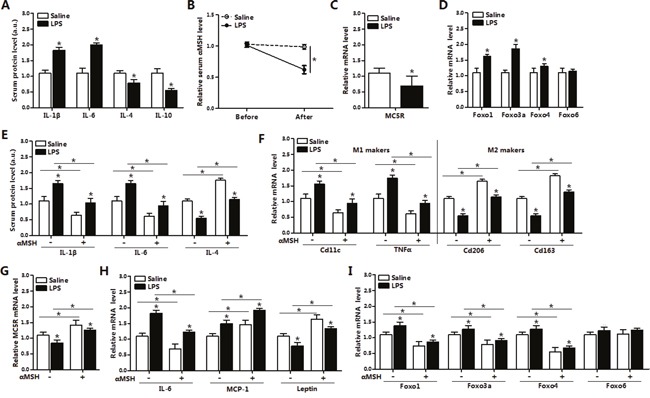
αMSH weakens LPS-induced adipose inflammation by inhibiting FoxOs expressions in mice **(A)** Serum protein levels of IL-1β, IL-6, IL-4 and IL-10 after mice with LPS or saline intraperitoneal injection (n=6). **(B)** Measurement of serum αMSH level before and after LPS or saline intraperitoneal injection (n=6). Epididymis white adipose tissue was isolated after intraperitoneal injection with LPS or saline, then mRNA levels of *MC5R* **(C)**, *Foxo1*, *Foxo3a*, *Foxo4* and *Foxo6* **(D)** were detected (n=6). Base on the LPS/saline injection, mice were injected with another 500 nM αMSH, then further detected for relative serum protein levels of IL-1β, IL-6, IL-4 **(E)** and mRNA levels of M1, M2 markers **(F)**, *MC5R* **(G)**, *IL-6*, *MCP-1*, *Leptin* **(H)**, *Foxo1*, *Foxo3a*, *Foxo4* and *Foxo6* **(I)** in white adipose tissue (n=6). Values are means ± SD. vs. control group, * *P* < 0.05.

To investigate the effects of αMSH on LPS-induced inflammation, we intraperitoneally injected LPS into male mice. We noted that LPS significantly increased the serum protein levels of pro-inflammatory cytokines IL-1β and IL-6 (*P*<0.05), while decreased the levels of anti-inflammatory cytokines IL-4 and IL-10 (*P*<0.05) (Figure 1A), indicating that LPS-induced inflammation model was successfully established. LPS decreased the serum level of αMSH and the mRNA expression of its receptor *MC5R* by approximately 45% (*P*<0.05) (Figure 1B and 1C). We observed significant increases in *Foxo1*, *Foxo3a* and *Foxo4* mRNA levels of white adipose tissue (*P*<0.05), but no effects on Foxo6 mRNA level (Figure 1D). In LPS-induced inflammation model, injection of αMSH significantly decreased mice serum protein levels of IL-1β and IL-6 (*P*<0.05), but increased the level of IL-4 (*P*<0.05) (Figure 1E). In addition, αMSH markedly decreased the mRNA levels of *IL-6*, *MCP-1* and M1 macrophage makers *CD11c* and *TNFα* (*P*<0.05), while increased the mRNA levels of *Leptin* and M2 macrophage makers *CD206* and *CD163* (*P*<0.05) (Figure 1F and 1H). These results indicated that αMSH weakened LPS-induced adipose inflammation. To investigate the mechanism by which αMSH exert this function, the expression of αMSH receptor MC5R and members in FoxOs family were detected. In accordance with the trend in inflammatory cytokines, αMSH reversed the mRNA level of *MC5R* reduced by LPS (*P*<0.05) (Figure 1G). Meanwhile, αMSH inhibited the mRNA levels of *Foxo1*, *Foxo3a* and *Foxo4* induced by LPS (*P*<0.05) (Figure 1I). These data indicated that αMSH blocked LPS-induced adipose inflammation in mice by inhibiting FoxOs expressions.

### FoxOs reverse the inhibition of αMSH on adipose inflammation in mice

**Figure 2:**
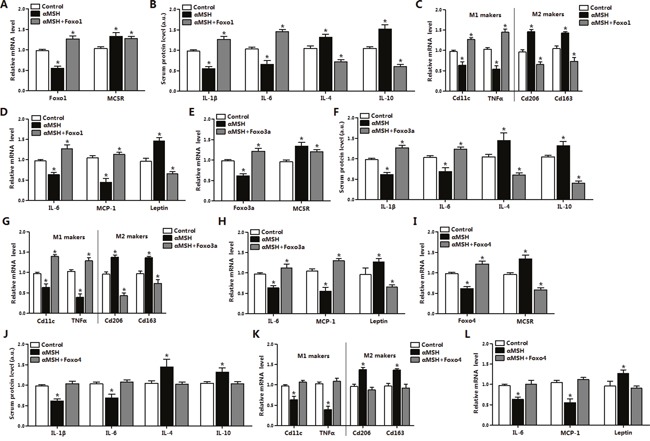
FoxOs reverse the inhibition of αMSH on adipose inflammation in mice Mice white adipose tissue was isolated after intraperitoneal injection with pAd-Foxo1 and with another 500 nM αMSH, then mRNA levels of *Foxo1*, *MC5R* **(A)**, M1, M2 markers **(C)**, *IL-6*, *MCP-1*, *Leptin* **(D)** were measured (n=6). After intraperitoneal injection with pAd-Foxo1 and with another 500 nM αMSH, serum protein levels of IL-1β, IL-6, IL-4 and IL-10 **(B)** were detected (n=6). After intraperitoneal injection with pAd-Foxo3a and with another 500 nM αMSH to mice, mRNA levels of *Foxo3a*, *MC5R* **(E)**, M1, M2 markers **(G)**, *IL-6*, *MCP-1*, *Leptin* **(H)** were measured in white adipose tissue (n=6). Serum protein levels of IL-1β, IL-6, IL-4 and IL-10 **(F)** were also detected (n=6). Mice were intraperitoneal injected with pAd-Foxo4 and another 500 nM αMSH, serum protein levels of IL-1β, IL-6, IL-4 and IL-10 **(J)** and mRNA levels of *Foxo4*, *MC5R* **(I)**, M1, M2 markers **(K)**, *IL-6*, *MCP-1*, *Leptin* **(L)** were measured (n=6). Values are means ± SD. vs. control group, * *P* < 0.05.

To further investigate how FoxOs act on αMSH inhibited adipose inflammation, we injected pAd-Foxo1, pAd-Foxo3a or pAd-Foxo4 to mice and then treated with hormone αMSH. It was observed that αMSH reduced and pAd-Foxo1 lifted *Foxo1* mRNA level in mice adipocytes as expected (*P*<0.05) (Figure 2A). αMSH individually enhanced the mRNA level of its receptor *MC5R* (*P*<0.05), which was slightly inhibited after pAd-Foxo1 treatment. In mice serum, we found decreases in IL-1β and IL-6 contents (*P*<0.05), while increases in IL-4 and IL-10 contents after αMSH treatment (*P*<0.05), but these effects were reversed by Foxo1 (*P*<0.05) (Figure 2B). Moreover, αMSH decreased the mRNA levels of M1 makers *CD11c* and *TNFα* (*P*<0.05), increased the levels of M2 makers *CD206* and *CD163* (*P*<0.05), and pAd-Foxo1 treatment reversed the effects (*P*<0.05) (Figure 2C). Notably, Foxo1 overexpression inverted the decrease of *IL-6* and *MCP1* mRNA levels, and the increase of *Leptin* level stimulated by αMSH (*P*<0.05) (Figure 2D). To determine whether other factors of FoxOs in adipose tissue have same effects, we also measured mRNA or serum protein level of inflammation factors, M1 makers and M2 makers after treating with pAd-Foxo3a and pAd-Foxo4, respectively. We observed Foxo3a and Foxo4 also reversed the inhibitory effects of αMSH on adipose inflammation, and the trend was same with Foxo1 (Figure 2E-2L). These data collectively suggested that Foxo1, Foxo3a and Foxo4 abolished the inhibition of αMSH on adipose inflammation in mice.

### FoxOs abolish the suppression of αMSH on inflammation in mice adipocytes

**Figure 3:**
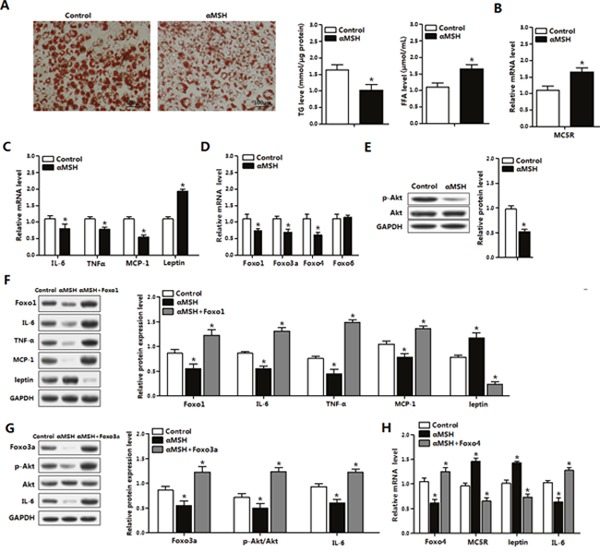
FoxOs abolish the suppression of αMSH on inflammation in mice adipocytes **(A)** Oil Red O staining for differentiated primary adipocytes isolated from epididymal white adipose tissue after administration of αMSH for 1 h (left). Relative concentration of TG in adipocytes (middle) and FFA in cell culture medium (right) were detected (n=3). With αMSH treatment, mRNA levels of *MC5R* (B), *IL-6*, *TNFα*, *MCP-1* and *Leptin* **(C)**, *Foxo1*, *Foxo3a*, *Foxo4* and *Foxo6* **(D)**, protein levels of p-AKT and total Akt **(E)** were detected in adipocytes (n=3). **(F)** Adipocytes treated with pAd-Foxo1 and αMSH, protein levels of IL-6, TNFα, MCP-1 and Leptin were measured (n=3). **(G)** Expression levels for Foxo3a, p-Akt, Akt and IL-6 protein after cells treated with pAd-Foxo3a and αMSH (n=3). **(H)** Normalized mRNA levels of *Foxo4*, *MC5R*, *Leptin* and *IL-6* with pAd-Foxo4 and αMSH treatments (n=3). Values are means ± SD. vs. control group, * *P* < 0.05.

Red O staining revealed that αMSH inhibited adipocyte differentiation, coordinated with triglycerides (TG) decreased and free fatty acid (FFA) increased (*P*<0.05) (Figure 3A). In differentiated adipocytes, αMSH elevated mRNA levels of its receptor *MC5R* and *Leptin* (*P*<0.05), while reduced the mRNA levels of *IL-6*, *MCP-1*, *TNFα*, *Foxo1*, *Foxo3a* and *Foxo4* in adipocytes (*P*<0.05) (Figure 3B-3D). Western blot analysis showed Foxo1 overexpression reversed the increased protein levels of Leptin and the deceased protein levels of Foxo1, IL-6, MCP-1 and TNFα treated by αMSH (*P*<0.05) (Figure 3F). To determine which signal pathway is involved in this process, we measured the phosphorylation level of Akt in adipocytes. We observed that αMSH significantly decreased Akt phosphorylation level (*P*<0.05) (Figure 3E), which is consistent with our previous findings that activation of Akt2 signal blunted RES inhibitory adipose inflammation [32]. Moreover, Foxo3a rescued the decreases of Foxo3a, IL-6 and p-Akt^Ser473^ protein levels treated with αMSH (*P*<0.05) (Figure 3G). Furthermore, Foxo4 only reversed the upregulation of *MC5R* and *Leptin* (*P*<0.05), and the downregulation of *Foxo4* and *IL-6* by αMSH in mRNA level (*P*<0.05) (Figure 3H), suggesting that αMSH may inhibit adipocyte inflammation partly via Foxo4 transcriptional regulation. These data indicated that Foxo1, Foxo3a and Foxo4 abolished the inhibition of αMSH on inflammation in mice adipocytes.

### Foxo1 and Foxo3a reverse the inhibition of αMSH on LPS-induced inflammation in mice adipocytes

**Figure 4:**
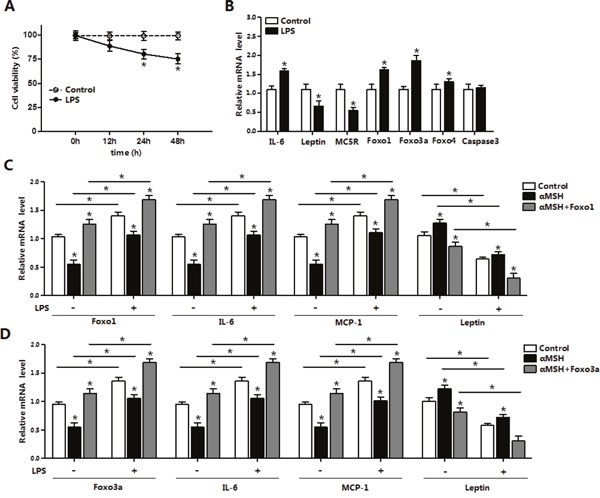
Foxo1 and Foxo3a reverse the inhibition of αMSH on LPS-induced inflammation in mice adipocytes **(A)** Primary adipocytes were cultured and incubated for 0 h, 12 h, 24 h and 48 h in the presence of 1 μg/mL LPS. Cell viability was detected by CCK-8 (n=3). **(B)** Relative mRNA expressions of *IL-6*, *Leptin*, *MC5R*, *Foxo1*, *Foxo3a*, *Foxo4*, *Foxo6* and *Caspase3* with αMSH treatment were analyzed (n=3). **(C)** After adipocytes treated with pAd-Foxo1 and incubated for LPS/saline and αMSH, mRNA levels of *Foxo1*, *IL-6*, *MCP-1* and *Leptin* were detected (n=3). **(D)** Adipocytes were treated with pAd-Foxo3a and incubated for LPS/saline and αMSH, then measured mRNA levels of *Foxo3a*, *IL-6*, *MCP-1* and *Leptin* (n=3). Values are means ± SD. vs. control group, * *P* < 0.05.

After 24 h and 48 h administration for LPS, live adipocytes number were significantly declined (*P*<0.05) (Figure 4A). LPS strikingly reduced the mRNA levels of *MC5R* and *Leptin* (*P*<0.05), while increased the levels of *IL-6*, *Foxo1*, *Foxo3a* and *Foxo4* (*P*<0.05), but did not change the expression of *Caspase3*, implying no adipocyte apoptosis occurrence (Figure 4B). LPS promoted the mRNA levels of *IL-6* and *MCP-1* (*P*<0.05), and inhibited *Leptin* mRNA expression (*P*<0.05) (Figure 4C and 4D). Furthermore, αMSH significantly decreased the mRNA levels of *IL-6* and *MCP-1* (*P*<0.05), increased the level of *Leptin* (*P*<0.05), but these effects were abolished by Foxo1 or Foxo3a (*P*<0.05) (Figure 4C and 4D). Together, these results revealed that αMSH can finely block the LPS induced inflammation and Foxo1, Foxo3a reversed the anti-inflammatory function of αMSH in mice adipocytes.

### Foxo4 attenuates MC5R transcription in αMSH inhibited inflammation in mice adipocytes

**Figure 5:**
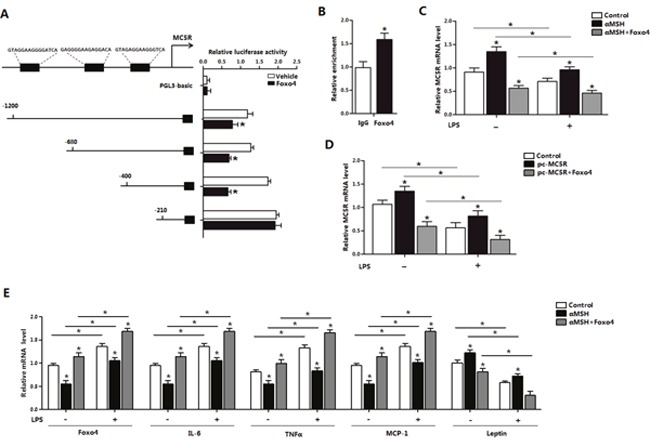
Foxo4 negatively regulate MC5R transcription in αMSH inhibited inflammation in mice adipocytes **(A)** Fragments of MC5R promoter fused to a luciferase reporter plasmid or PGL3-basic (control) were co-transfected into cells together with Renlilla plasmid and pAd-Foxo4 (n=3). Luciferase activity was corrected for Renilla luciferase activity and normalized to control activity (n=3). **(B)** Chromatin immunoprecipitation (ChIP) analysis of Foxo4 and MC5R interaction. **(C, D)** After pAd-Foxo4 together with αMSH or pc-MC5R in LPS/saline treatment, *MC5R* mRNA level was determined in adipocytes (n=3). **(E)** When adipocytes were treated with pAd-Foxo4 and αMSH in LPS/saline treatment, mRNA levels of *Foxo4*, *IL-6*, *TNFα*, *MCP-1* and *Leptin* were analyzed (n=3). Values are means ± SD. vs. control group, * *P* < 0.05.

To explore how Foxo4 influence the transcription of MC5R in αMSH inhibited inflammation, we detected the binding affinity between Foxo4 and MC5R promoter. Luciferase assay was carried out after co-transfection of Foxo4 overexpression vector and plasmid with different MC5R 5′regions in HEK393T cells, and results showed that the luciferase activity of MC5R was declined on the sites of -1200, -680, -400 bp, but not -210 bp when Foxo4 overexpressed (*P*<0.05) (Figure 5A). Enrichment of Foxo4 also significantly increased in Foxo4 overexpressed group (*P*<0.05) (Figure 5B), suggesting that Foxo4 binds to MC5R promoter. LPS inhibited *MC5R* mRNA expression (*P*<0.05), however, αMSH treatment or overexpressed MC5R plasmid transfection both significantly increased MC5R level in non LPS and LPS-induced condition (*P*<0.05), and these effects was reversed by Foxo4 overexpression (*P*<0.05) (Figure 5C and 5D). LPS significantly increased the mRNA levels of *Foxo4*, *IL-6*, *TNFα* and *MCP-1* (*P*<0.05), decreased the level of *Leptin* (*P*<0.05) (Figure 5E). αMSH decreased the mRNA levels of *Foxo4*, *IL-6*, *TNFα* and *MCP-1* (*P*<0.05), increased the level of *Leptin* (*P*<0.05), but Foxo4 reversed the effects (*P*<0.05) (Figure 5E). These data suggested that αMSH inhibited LPS-induced inflammation in mice adipocytes through Foxo4 negative transcription on MC5R promoter.

### Akt/JNK signal pathway is impaired in the inhibition of αMSH on adipocyte inflammation and FoxOs expressions

**Figure 6:**
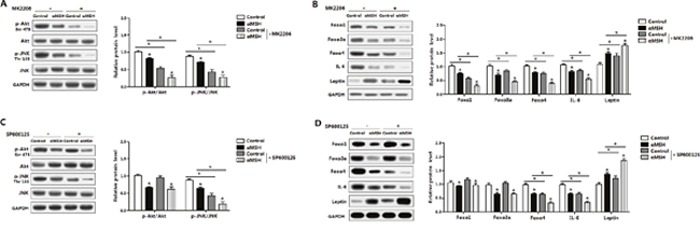
Akt/JNK signal pathway is impaired in the inhibition of αMSH on adipocyte inflammation and FoxOs expressions Mouse adipocytes were pretreated with αMSH and MK-2206 or SP600125, respectively. Relative protein levels of Akt, p-Akt^ser473^, JNK, p-JNK^Thr183^ **(A)**, Foxo1, Foxo3a, Foxo4, IL-6 and Leptin **(B)** with or without MK-2206 (n=3). Representative immunoblots and densitometric quantification for Akt, p-Akt^ser473^, JNK, p-JNK^Thr183^ **(C)**, Foxo1, Foxo3a, Foxo4, IL-6 and Leptin protein **(D)** with or without SP600125 (n=3). The level of total GAPDH was used as the loading control. Values are mean ± SD. *P < 0.05 compared with the control.

To further elucidate molecular mechanism in αMSH inhibited adipocyte inflammation, we analyzed the major inflammatory pathways in adipose tissue. Figure 6A showed αMSH treatment in adipocytes decreased the phosphorylation level of Akt^Ser473^ and JNK^Thr183^ (*P*<0.05). When the special Akt inhibitor MK-2206 was added, phosphorylation levels of Akt^Ser473^ and JNK^Thr183^ were correspondingly decreased (*P*<0.05), and αMSH treatment further decreased these protein levels (*P*<0.05) (Figure 6A), which agree with our previous study that the Akt2 pathway was significantly activated when adipose inflammation happened [32]. The addition of αMSH also aggravated the elevation on Leptin protein level and the inhibition of Foxo1, Foxo3a, Foxo4 and IL-6 treated with MK-2206 (*P*<0.05) (Figure 6B). To further identify whether other signaling pathways regulating adipocyte inflammation may exist downstream of Akt, we used the special JNK inhibitor SP600125 to treat cells. We observed that SP600125 significantly inhibited phosphorylation of JNK^Thr183^ (*P*<0.05), but had no effects on phosphorylation of Akt^Ser473^ (Figure 6C), suggesting that JNK signaling pathway may act downstream of Akt pathway, which is coherent with previous findings [33]. In addition, SP600125 decreased the protein levels of Foxo4 and IL-6 (*P*<0.05), increased Leptin protein level (*P*<0.05), but had no effects on the expressions of Foxo1 and Foxo3a (Figure 6D). αMSH further reduced the protein levels of p-Akt^Ser473^, p-JNK^Thr183^, Foxo4 and IL-6 (*P*<0.05), while promoted Leptin protein level (*P*<0.05) (Figure 6C and 6D). This revealed that Foxo4 but not Foxo1 and Foxo3a reversed the effects of αMSH on adipocyte inflammation through JNK pathway. Thus, these data supported that αMSH inhibited adipocyte inflammation by blocking Akt/JNK signaling pathway and FoxOs expressions.

## DISCUSSION

αMSH as a tridecapeptide derived from POMC displays potent anti-inflammatory role in many tissues and has protective effects on therapies in brain damage, liver or lung fibrosis, skin inflammation and chronic adipose inflammation. The fusion protein TAT-HSA-α-MSH inhibits NF-κB activation in human and TNFα production in mice to prevent brain inflammation in central nervous system (CNS) disorders [34, 35]. αMSH has also been confirmed to inhibit antigen-induced allergic skin inflammation, abolish monocytes adhesion to vascular endothelium, attenuate bleomycin-induced pulmonary inflammation, blunt adipose oxidative stress and inflammation, and protect from adipose tissue apoptosis and fat deposition [24, 36–40]. In the present study we illustrated that αMSH could blunt LPS-induced adipose inflammation, accompany with increased anti-inflammatory cytokines expressions and decreased pro-inflammatory cytokines expressions. Moreover, αMSH attenuated adipose inflammation in mice by inhibiting FoxOs expressions. Although Jun et al. reported that αMSH in addition to Adrenocorticotropic hormone (ACTH) promotes adipocyte inflammation by cytokine IL-6 production [41], most studies confirmed that αMSH resists to adipose inflammation by releasing anti-inflammatory factors and balancing metabolic rate [24, 42, 43], which are consistent with our findings.

**Figure 7:**
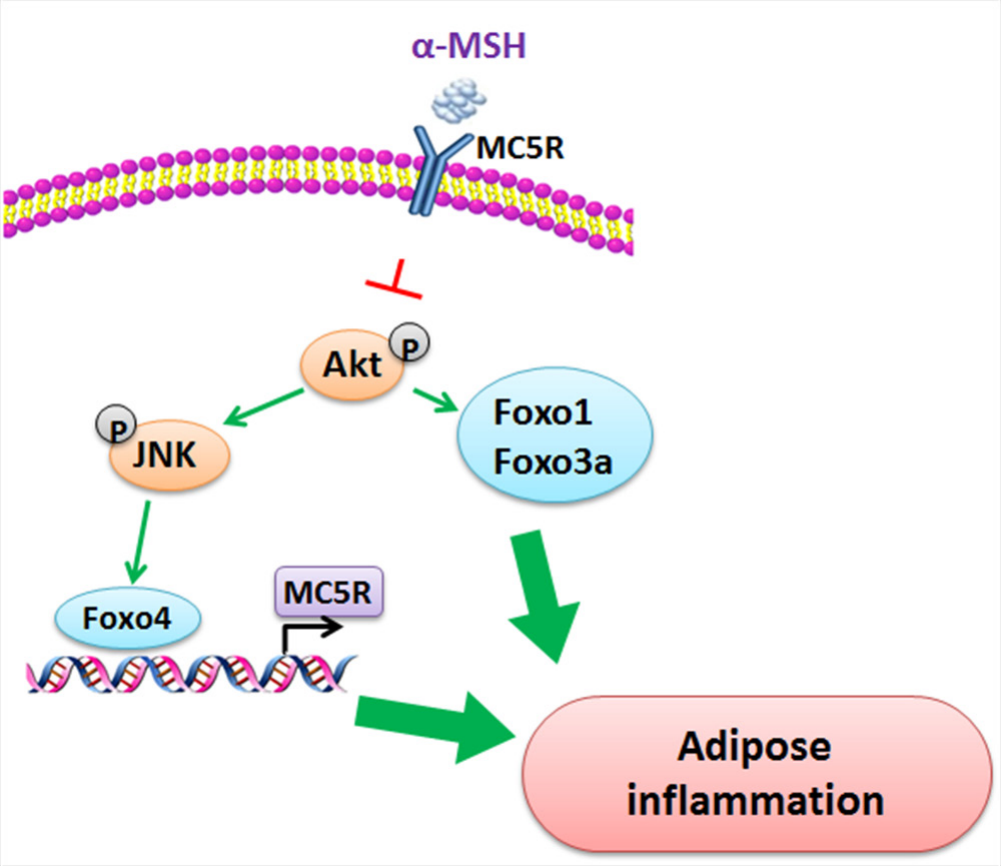
Proposed mechanism of the inhibition of αMSH on adipose inflammation αMSH decreases adipose inflammation via blunting the phosphorylation of Akt/JNK. Akt phosphorylation activates Foxo1 and Foxo3a, while JNK phosphorylation activates Foxo4. What is more, Foxo4 acts via binding the promoter of MC5R, which is the receptor of αMSH.

FoxOs in mammals are important transcription factors in regulating adipose inflammation. Our results showed a significant increase of mRNA levels of Foxo1, Foxo3a and Foxo4 in adipose tissue induced by LPS, but had no effects on Foxo6, so we focused on the effects of Foxo1, Foxo3a and Foxo4 on αMSH inhibited adipose inflammation. Studies have shown that phosphorylation of Foxo1 and Akt^Ser473^ is activated in endothelial nitric oxide synthase (eNOS) production, which promotes adipose inflammatory reaction [44]. Pdk1-Foxo1 signaling also contributes to adipose inflammation and insulin resistance [45]. For Foxo3a, it has been reported that SIRT1-FoxO3A axis acts on autophagy activation in macrophages [46] and Foxo3a is also required in inflammation to prevent Fas ligand-induced neutrophil apoptosis [47]. Our results proved that Foxo1 and Foxo3a both aggravated LPS-induced inflammation in mice adipocytes, plus Foxo1, Foxo3a and Foxo4 reversed the inhibition of αMSH on adipose inflammation *in vivo* and *in vitro*. For another, Foxo4 is confirmed to be the center of a transcriptional regulatory network that links gene transcription required for many inflammatory cytokine signals [29, 48, 49]. We showed here Foxo4 negatively regulated MC5R promoter transcription, which brought to the abolition of αMSH's anti-inflammation effects. These results demonstrate FoxOs are aggressive factors in adipose tissue inflammation. When further study the signal pathway which αMSH interacts with, we examined the influence of Akt/JNK signal pathway, a key pathway in adipose inflammation [32]. Our study revealed that αMSH could blunt the phosphorylation of Akt/JNK and specifically, Akt phosphorylation activates Foxo1 and Foxo3a, while JNK phosphorylation stimulates Foxo4. In addition, Foxo4 acts via binding the promoter of MC5R, which is the receptor of αMSH. (Figure 7). These findings are coherent with the previous study, which show FoxOs are required for Akt activity and JNK signaling pathway in systemic inflammation and cells survival [50, 51].

In summary, our study provided a new insight into the mechanisms of αMSH on adipose inflammation. We identified that αMSH inhibited adipose inflammation by inhibiting Akt and JNK signaling pathway and FoxOs expressions. Moreover, we found that Foxo4 was a novel transcriptional factor of MC5R in aggravating adipose inflammation. These results will be valuable for prevention and treatment of inflmmatory diseases.

## MATERIALS AND METHODS

### Animal experiments

Two-week-old male Kunming mice were purchased from the Laboratory Animal Center of the Fourth Military Medical University. All mice were treated in accordance with the applicable guidelines and regulations approved by the Animal Ethics Committee of Northwest A&F University. They were allowed ad libitum access to water and standard chow laboratory diet. The animal room was maintained in controlled conditions of temperature at 25 ± 1 °C, humidity at 55 ± 5%, and a 12 h light/12 h dark cycle. Body weight was recorded once a week. After intraperitoneal injection of pAd-Foxo1, pAd-Foxo3a, pAd-Foxo4 for 10 d and 500 nM αMSH pre-treated for 1 h, mice were euthanized for collection of tissues and blood.

### Primary cell culture

Epididymal white adipose tissues were harvested from four-week-old mice, visible fibers and blood vessels were removed, and the adipose tissue was washed three times with PBS buffer containing 200 U/mL penicillin (Sigma) and 200 U/mL streptomycin (Sigma). The adipose tissue was minced into fine sections (1 mm^3^) and incubated in 10 mL digestion buffer containing DMEM/F-12 (Gibco), 100 mM HEPES (Sigma), 1.5% BSA (Sigma), and 2 mg/mL type I collagenase (Sigma) for 50 min at 37 °C water bath. After the incubation, growth medium [DMEM/F-12 (50:50)], 10% fetal bovine serum (Sigma), 100 U/mL penicillin, and 100 U/mL streptomycin were added to the digestion flask. Flask contents were mixed and filtered through nylon screens with 250 μm and 20 μm meshes to remove undigested tissue and large cell aggregates. The filtered cells were centrifuged at 1300 rpm for 7 min to separate floating adipocytes from cell pellets. Isolated cell pellets were suspended in DMEM/F12 (Invitrogen). Finally, cells were seeded into 35 mm primary culture dishes at a density of 8 × 10^4^ cells/dish and incubated at 37 °C under a humidified atmosphere of 5% CO_2_ and 95% air until confluence.

### Chemical treatment and transfection

The recombinant adenovirus overexpression vector of Foxo1 (pAd-Foxo1), recombinant adenovirus overexpression vector of Foxo3a (pAd-Foxo3a), recombinant adenovirus overexpression vector of Foxo4 (pAd-Foxo4), recombinant adenovirus overexpression vector of Foxo6 (pAd-Foxo6) and overexpression plasmid vector of MC5R (pc-MC5R) were constructed in our lab. When primary adipocytes were plated at a concentration of 1×10^5^-1×10^6^/ml in 60 mm dish (suspension cells), these recombinant adenovirus vectors were respectively mixed with Opti-MEMI media (Invitrogen) and X-treme GENE HP Reagent (Roche, Switzerland), then the mixture were added to each culture dishes afterwards. The αMSH (Sigma, St. Louis, MO, USA) and the working solution (500 nM) were mixed to treat adipocytes for 1 h. Cells were collected for Real-time PCR after 24 h infection and for Western blot after 48 h.

On the fourth day of cell differentiation, cells were treated with 10 μM Akt phosphorylation-specific inhibitor MK-2206 (MedChem Express, USA), 10 μM JNK phosphorylation-specific inhibitor SP600125 (Selleck Chemical, USA), respectively. The αMSH (Sigma, St. Louis, MO, USA) was added to adipocytes for 1 h before collecting for Western blot analysis.

### LPS-induced administration

Inflammation model was established by intraperitoneal (i.p.) injection of LPS in 0.9% saline at a dose of 1 mg/kg of body weight in mice. Control mice were injected with saline only. Experiments were performed at 24 h after LPS administration.

*In vitro*, primary adipocytes isolated from eWAT were cultured to a concentration of 1×10^5^-1×10^6^/ml in 60 mm dish, and then incubated with 1 μg/mL LPS. Control group was treated with PBS. Cell viability was measured after incubating for 0 h, 12 h, 24 h and 48 h. Gene expression profile was analyzed at 24 h after LPS treatment.

### Oil red O staining

Lipid droplets were stained with Oil Red O staining as described in our previous publication [32]. Firstly, primary adipocytes were washed with PBS thrice, and then incubated in 4% formaldehyde for 30 minutes at room temperature. Then cells were gently washed with PBS thrice, stained with Oil Red O (Sigma, St. Louis, USA) for 30 min, and incubated at 37 °C temperature. Cells were washed in PBS for 3 times to remove unbounded dye, and then took photographs with Nikon TE2000-S florescent microscope. The stained fat droplets were dissolved in isopropanol and quantified by measuring the optical density at 510 nm using a spectrophotometer. Total protein content was measured with the DC assay (BioRad, USA) and triglycerides were analyzed by TG assay kit (Jiancheng, China). Free fatty acid (FFA) content was determined at absorption at 570 nm with a FFA assay kit (Jiancheng, China).

### ELISA assay

Mice blood supernatants were collected and frozen at -80 °C. αMSH content and serum protein levels of IL-1β, IL-4, IL-6 and IL-10 were measured using commercial ELISA kits (R&D Systems, USA), the procedure was described previously [52].

### Real-time PCR

Total RNA was extracted with TRIpure Reagent kit (Takara, China). 500 ng of total RNA was reverse transcribed using M-MLV reverse transcriptase kit (Roche). Primers of *CD11c*, *TNFα*, *CD206*, *CD163*, *MC5R*, *IL-6*, *MCP-1*, *Leptin*, *Foxo1*, *Foxo3a*, *Foxo4*, *Foxo6*, *Caspase3* and *GAPDH* were designed as Table 1 and synthesized by Invitrogen. Quantitative PCR was performed in 20 μL reaction system containing specific primers, SYBR Premix EX Taq (Takara, China), cDNA and ddH_2_O. The level of mRNA was normalized by *GAPDH*. The relative mRNA levels of genes were analyzed with the method of 2^-ΔΔCt^.

**Table 1:** Primers for real-time PCR

Genes	Accession number	Primer sequences (5' to 3')
CD11c	NM_021334.2	F: ATGATAGTTCCTGGGTGGTGGTTGG
		R: AGAGAACTGCATCAGGGAGAACCGT
TNFα	NM_013693.3	F: ACGGCATGGATCTCAAAGAC
		R: CGGCAGAGAGGAGGTTGACT
CD206	NM_008625.2	F: CTCTGTTCAGCTATTGGACGC
		R: TGGCACTCCCAAACATAATTTGA
CD163	NM_001170395.1	F: GGTGGACACAGAATGGTTCTTC
		R: CCAGGAGCGTTAGTGACAGC
MC5R	NM_013596.2	F: CAAGACCAGAGCCCGGTAAAC
		R: GCGCAAAGGTAAGCATGATTCT
IL-6	NM_031168.2	F: AGACAAAGCCAGAGTCCTTCAG
		R: TGCCGAGTAGATCTCAAAGTGA
MCP-1	NM_011333.3	F: CACAACCACCTCAAGCAC
		R: AAGGGAATACCATAACATCA
Leptin	NM_008493.3	F: GAAGCGTCTCGGGATCTCTG
		R: CAGGATCAATGACATTTCACACA
Caspase3	NM_009810	F: CTCGCTCTGGTACGGATGTG
		R: TCCCATAAATGACCCCTTCATCA
Foxo1	NM_019739.3	F: GGACAGCCGCGCAAGACCAG
		R: TTGAATTCTTCCAGCCCGCCGA
Foxo3a	NM_019740.2	F: GTGGACCGACTTCCGCTCGC
		R: GCTTGCCAGGATGGGCGACA
Foxo4	NM_018789.2	F: ACTTTGAGCCAGATCCCTGAGTCAC
		R: TAAGGACAGGCCTGGCTCCACC
Foxo6	NM_194060.1	F: GTGGGGGAACCTTTCCTACG
		R: TTCTGCACGCGGATGAACC
GAPDH	NM_001289726.1	F: AGGTCGGTGTGAACGGATTTG
		R: TGTAGACCATGTAGTTGAGGTCA

### Immunoblot analysis

Mouse adipocytes were solubilized in lysis buffer for 40 min at 4 °C, then the solution was centrifuged at 12,000 g for 15 min at 4 °C and the supernatants were to determine protein concentration. In addition, protein samples (30μg) were separated by electrophoresis on 12% and 5% SDS-PAGE gels using slab gel apparatus, and transferred to PVDF nitrocellulose membranes (Millipore, USA) blocked with 5% skim milk powder/Tween 20/TBST at room temperature for 2 h. The membranes were then incubated with with primary antibodies in 5% milk overnight at 4°C. Foxo1 (ab52857), Foxo3a (ab17026), Foxo4 (ab63254), IL-6 (ab7737), TNFα (ab9739), MCP-1 (ab25124), JNK (ab199380), JNK (Thr183) (ab47337) antibodies were purchased from Abcam (Cambridge, UK). Akt (BS1978), phospho-Akt (Ser473) (BS4006), GAPDH (Ap0063) antibodies were purchased from Bioworld (Nanjing, China). Leptin (sc-9014) was purchased from Santa Cruz Biotechnology (CA, USA). Followed, the appropriate HRP conjugated secondary antibodies (Boaoshen, China) were added and incubated for 2 h at room temperature. Proteins were visualized using chemiluminescent peroxidase substrate (Millipore), and then the blots were quantified using ChemiDoc XRS system (Bio-Rad, USA).

### Luciferase reporter assays

Four fragments containing MC5R-5’ sequences from -1200 to -210 relative to the transcription initiation site were sub-cloned into pGL3-basic vector (Takara, China). Luciferase reporter assay procedure was performed as previously described [53]. Briefly, HEK293T cells were cultured in 24-well plates. Cells were co-transfected with Foxo4 overexpression vector, Renilla plasmid and pGL3-MC5R plasmid. pGL3-basic vector was considered as control reporter. Cells were harvested 48 h after transfection, and detected using the Dual-Luciferase Reporter assay system (Promega, USA). Luciferase activity was obtained by an average of luciferase assay experiments at least three times.

### Statistical analysis

Statistical calculations were performed with SAS v8.0 (SAS Institute, Cary, NC). The effects of experimental treatments were determined using the one-way ANOVA procedure. Comparisons among the means of individual treatments were made by Fisher's least significant difference (LSD) post hoc test once the ANOVA analysis showed a significant effect of these treatments. Data are presented as mean ± SD and from three independent experiments. *P < 0.05 was considered to be significant.
